# Social and cultural determinants of traditional, complementary and integrative medicine (TCIM) use in primary care: the role of migration, health literacy, and religious orientation

**DOI:** 10.1186/s12889-026-29033-1

**Published:** 2026-08-26

**Authors:** G. Ciarlo, J. Buentzel, S. Grüll, J. Huebner

**Affiliations:** 1 Medizinische Klinik I, Universitätsklinikum Frankfurt, Theodor-Stern-Kai 7, Frankfurt am Main, 60590 Germany; 2 Department of Otorhinolaryngology, Palliative Care Unit, Südharz Klinikum Nordhausen, Dr.-Robert-Koch-Straße 39, Nordhausen, 99734 Germany; 3 Klinik für Innere Medizin II, Universitätsklinikum Jena, Am Klinikum 1, Jena, 07747 Germany; 4 Medical Clinic 1, Gastroenterology, hepatology, pneumology, allergology, nutritional medicine, endocrinology, diabetology, University Hospital Frankfurt, Theodor-Stern-Kai 7, Frankfurt am Main, 60590 Germany

**Keywords:** Religious orientation, Complementary medicine use, TCIM, Health literacy, Migrant healthcare

## Abstract

**Background:**

This study examines whether migration background, health literacy, and religious–spiritual orientation are associated with the use of traditional, complementary and integrative medicine (TCIM). In addition, the relationship between religious–spiritual orientation, Religious Orientation (RO), and different TCIM modalities was explored. From a public health perspective, migration background, health literacy, and social determinants may further influence patterns of traditional, complementary and integrative medicine (TCIM) use. This study examines how religious orientation relates to the use of body–mind and biological TCIM approaches and whether these dimensions function as coping resources or determinants of health behaviour.

**Methods:**

Between October 2023 and January 2024, 375 patients were recruited in a single-center primary care study. Data were collected using an anonymous questionnaire assessing TCIM use, health literacy, religious orientation, and sociodemographic characteristics.

**Results:**

Of all participants, 19.5% reported a migration background. The most frequently used TCIM methods were physical activity (75.2%), vitamins and minerals (41.6%), and vitamin D (35.6%). Overall TCIM use was largely comparable between participants with and without a migration background, with a significant difference observed only for homeopathy (14.1% vs. 6.1%, *p* = 0.007). Participants with a migration background showed higher religious orientation scores, while specific RO were determined by religious affiliation. Health literacy levels were generally high and varied mainly by age, gender, and education, showing only weak associations with TCIM use. Female gender and higher educational level were the most consistent predictors of TCIM use.

**Conclusion:**

TCIM use was common in primary care patients and showed little association with migration background or religious orientation, despite higher religiosity among migrants. Religiosity appears to function primarily as a psychosocial coping resource rather than a determinant of therapeutic behavior. From a public health perspective, TCIM use appears to be more strongly shaped by gender and educational level than by migration background or religious orientation. Female participants consistently reported higher use of several TCIM modalities, while educational level was primarily associated with lifestyle-related approaches such as physical activity. These findings underscore the importance of addressing social determinants and avoiding culturally stereotypical assumptions in primary care.

## Key points

TCIM use was common among primary care patients and showed only minor differences between individuals with and without a migration background. Although participants with a migration background reported higher levels of Religious Orientation (RO), neither RO nor health literacy was consistently associated with specific TCIM modalities. Instead, female gender and educational level emerged as the most consistent predictors of TCIM use, while health literacy showed only selective, modality-specific associations.

## Introduction

Chronic illness imposes psychological and existential demands that vary across individuals and cultural contexts and often extend beyond the immediate somatic burden. Being faced with a long-term and potentially life-altering condition raises fundamental questions about meaning, fairness, and personal vulnerability. In the course of adapting to chronic disease, patients may experience different stages of psychological processing. At the beginning, patients may experience concern or insecurity, which can be accompanied by frustration or resistance. Over time, individuals often attempt to regain control through coping strategies, lifestyle adjustments, or seeking alternative perspectives. Feelings of sadness and withdrawal may occur before a phase of acceptance gradually sets in, marked by a more conscious and active approach to living with the illness [1]. These adaptive processes show considerable interindividual variability and do not necessarily follow a linear trajectory.

Coping responses to serious illness differ substantially between individuals and are shaped by personal resources as well as sociocultural context [2, 3]. Psychological adjustment mechanisms differ between individuals and are embedded within their broader cultural and social context [4]. Social networks, particularly close family members, frequently provide essential emotional and practical support during the adjustment process [5]. However, many affected individuals also find hope and comfort in spiritual or religious resources [6–8]. These resources may function as protective factors within a vulnerability-stress framework [9], in which individual predispositions interact with external stressors and available coping capacities to shape psychological outcomes. In the psychology of religion, the precise measurement of intrinsic and extrinsic orientations toward religiosity has been a central topic of debate for more than three decades. Various proposals have been made to refine the assessment of these dimensions, aiming to capture both the internalized, meaning-oriented aspects of faith and the more socially or instrumentally motivated expressions of religiosity.

Traditional, complementary and integrative medicine (TCIM) represents a relevant interface between spirituality, religious orientation, and health-related decision-making. According to the World Health Organization (WHO), traditional medicine refers to the knowledge, skills, and practices indigenous to different cultures used to maintain health and prevent, diagnose, and treat illness. Complementary medicine comprises health practices that are not part of a country’s conventional healthcare system, whereas integrative medicine refers to the coordinated use of conventional and complementary approaches in a person-centred manner. Practices such as meditation, yoga, mindfulness, guided imagery and breathing exercises offer an integrative approach by emphasizing the connection between mental and physical health and often incorporating spiritual aspects [9]. These approaches may contribute to stress reduction while promoting inner stability and self-efficacy.

The increasing integration of TCIM into the care of chronically and seriously ill patients in some healthcare settings underscores the relevance of subjective illness perceptions, cultural background, and individual beliefs in clinical decision-making [10]. In oncology in particular, TCIM is used predominantly as a complement to conventional cancer treatment rather than as a substitute. Patients most commonly use TCIM for symptom management, emotional support, and meaning-making alongside standard medical care [11, 12]. Nevertheless, there is still a lack of systematic research examining how these factors interact in culturally sensitive, patient-centred care.

The aim of this study was to investigate the use of traditional, complementary and integrative medicine (TCIM) among primary care patients and to examine whether migration background, health literacy, and religious orientation are associated with TCIM use. In addition, we explored the associations between these factors and different TCIM modalities to improve the understanding of social and cultural determinants of TCIM use in primary care.

## Patients and methods

### Study population

This prospective cross-sectional study was implemented at a single outpatient primary care center that delivers routine services to around 4,000 patients annually, including a markedly heterogeneous migrant community. Between October 2023 and January 2024, a consecutive convenience sample of adult patients attending the primary care practice was recruited. All eligible patients who presented during the recruitment period were invited to participate and completed an anonymized, structured questionnaire assessing TCIM use, health literacy, religious orientation, and sociodemographic characteristics. Six questionnaires contained incomplete data; therefore, the number of observations varies slightly across analyses depending on the availability of data for the respective variables. All participants received written study information and provided informed consent prior to participation. Recruitment and data collection were performed on site by trained staff following standardized procedures.

The sociodemographic and religious heterogeneity of this cohort provided an appropriate basis for examining the relationships between migration background, health literacy, religious orientation, and TCIM use.

### Inclusion and exclusion criteria

Eligibility required that patients were able to give informed consent and had sufficient language proficiency to complete the survey on their own or with the help of an interpreter. Those who did not fulfill these criteria were not considered for participation.

### Endpoints

The primary endpoint of this study was to examine whether migration background, health literacy, and religious orientation are associated with overall TCIM use among primary care patients.

Secondary analyses explored associations between religious orientation, health literacy, sociodemographic characteristics, and specific categories of TCIM use.

### Questionnaire

A structured survey instrument was designed to capture the interconnections between TCIM use, health literacy, and religious faith among patients with and without a migration background.

The survey encompassed five thematic domains:

1. *Sociodemographic and Health Profile* – including age, gender, country of origin, length of stay in Germany, residence, religion, education, and current or past illnesses. Migration background was defined as being born outside Germany or having at least one parent born outside Germany. For participants with a migration background, additional variables were collected, such as self-rated German proficiency, health status, and language use across different contexts (reading, speaking, thinking, media consumption, at home, and in social settings). Social preferences regarding friends, contacts, and children’s friendships were also documented.
2. *Acculturation* – measured with the 12-item SASH, covering language use in various contexts, media language preferences, and ethnic social affiliations. Items were rated on a 5-point Likert scale ranging from exclusive use of the mother tongue to exclusive use of German.
3. *Health Attitudes and Self-Management* – focusing on perceived responsibility for health, ability to prevent or manage illness, knowledge of treatments, confidence in lifestyle changes, and engagement in health-promoting behaviors. Responses were recorded on a 4-point Likert scale from “not true” to “true.”
4. *Trust in Physicians and Healthcare Satisfaction* – assessed through items on confidence in medical decisions, perceived thoroughness, reliability, and overall trust in physicians. Satisfaction was measured via communication quality, attentiveness, and patient-centeredness, using a 7-point Likert scale from “strongly disagree” to “strongly agree.” Scores were categorized into low (10–24), moderate (25–39), high (40–54), and very high (55–70).
5. *Religious Orientation (RO)*** –** Religious orientation was assessed using five investigator-developed items specifically created for this study rather than a previously validated questionnaire. The items reflected both intrinsic and extrinsic aspects of religious orientation and were combined into a composite measure of overall religious orientation as a single construct. The five-item composite demonstrated excellent internal consistency (Cronbach’s α = 0.92). The items addressed personal prayer and reflection, perceived divine presence, religious coping, and the role of religion in everyday life. As no validated religiosity questionnaire was used, internal consistency of the study-specific items was evaluated using Cronbach’s alpha. Responses were rated on a 4-point Likert scale ranging from “does not apply” to “fully applies.”
6. *Resilience* – participants self-assessed adaptive coping, stress regulation, persistence, flexibility, and emotional stability. Items were scored on a 5-point Likert scale from “not true at all” to “always true.”
7. *TCIM Use and Trust in Media Sources*: TCIM use was assessed using dichotomous items of the German version of the International Complementary and Alternative Medicine Questionnaire (I-CAM-G) [13], indicating current use (yes/no) of specific TCIM modalities. Although the questionnaire retains its original name, the term “traditional, complementary and integrative medicine (TCIM)” is used throughout this manuscript in accordance with current terminology. In the present survey, physical activity was included as a health-related lifestyle behavior because it is frequently recommended as part of integrative care and self-management strategies for chronic diseases. It should therefore be interpreted as a complementary health-related behavior rather than a classical TCIM modality.

As the assessed modalities represent conceptually distinct health practices rather than a single underlying construct, no composite TCIM score was calculated and no factor analysis was performed. Instead, TCIM use was analysed on three levels: (a) method-specific use of individual TCIM modalities, (b) a binary indicator reflecting any TCIM use (use of at least one modality), and (c) a categorical indicator of TCIM use breadth, distinguishing low use (1–2 modalities) from high use (≥ 3 modalities). Trust in traditional and social media was rated on a seven-point scale ranging from “no trust at all” to “very high trust.”

The questionnaire consisted of 72 items covering several health-related domains designed to comprehensively characterize the study population. These domains included sociodemographic characteristics, health literacy, trust in physicians and healthcare satisfaction, TCIM use, media trust, religious orientation, and resilience. The present analysis focuses on TCIM use and its associations with migration background, health literacy, and religious orientation.

### Statistical analysis

All analyses were conducted using IBM SPSS Statistics (version 31). Descriptive statistics (frequencies, percentages, means, standard deviations, and ranges) were used to characterize the study population and to summarize key variables, including TCIM use, health literacy, and Religious Orientation (RO).

Group differences between participants with and without a migration background, as well as between sociodemographic subgroups (age, gender, education), were examined using chi-square tests for categorical variables and independent-samples t-tests or one-way ANOVA for continuous variables. Ordinal trends across age and education groups were additionally assessed using non-parametric tests for trend.

Exploratory associations between Religious Orientation (RO), sociodemographic characteristics, and migration background were analysed using multinomial logistic regression models. Binary logistic regression analyses were performed for TCIM modalities with sufficient prevalence (e.g., vitamin D, other vitamins/minerals, medicinal plants, and physical activity) to identify predictors of use, including migration background, gender, age, and education. Model fit (Nagelkerke R²), classification accuracy, regression coefficients, odds ratios, and 95% confidence intervals were reported.

Exploratory analyses were also performed to examine whether RO and health literacy were associated with TCIM use. Bivariate chi-square tests and logistic regression models were applied. Internal consistency of the selected RO items was assessed using Cronbach’s alpha.

The primary analyses examined the associations between TCIM use and migration background, health literacy, religious orientation, and sociodemographic characteristics. Additional analyses were conducted within an exploratory framework, acknowledging the cross-sectional design, the heterogeneity of the sample, and the observational nature of the study. Summary variables for TCIM use (overall use as well as low versus high use) were derived post hoc from the individual TCIM items to capture different patterns of TCIM utilization. Analyses were performed using all available data for each variable (available-case analysis); consequently, the number of observations varied slightly across analyses because of missing responses. Given the exploratory nature of the secondary analyses, no formal adjustment for multiple comparisons was applied. These findings should therefore be interpreted as hypothesis-generating rather than confirmatory.

### Ethical vote

The study was carried out in line with the ethical standards set forth in the Declaration of Helsinki. Participation was voluntary and anonymous; completion and return of the questionnaire signified consent. Ethical review was obtained from two independent institutions: the Ethics Committee of the University Hospital Jena and, due to data collection in a rural primary care practice in Neudenau near Heilbronn, also from the Ethics Committee of the State Medical Association of Baden-Württemberg. Formal approval was granted on October 16, 2023 (Approval No. 2023-3129-Bef).

## Results

### Demographic data

Between October 2023 and January 2024, 375 patients participated in the study; 369 completed questionnaires were available for analysis. The mean age was 50.3 years (range 18–88), with 54.5% female and 45.2% male. Nearly one fifth (19.5%) reported a migration background, most lived in rural areas (96.2%), and the majority identified as Christian (71.2%). Migrants were more likely to be married and had more children on average than native-born (*p* < 0.05 and *p* < 0.01, respectively). The groups also differed in educational attainment, with non-migrant participants more likely to report higher qualifications and migrants more likely to report secondary school diplomas (*p* < 0.05). Demographic data are summarized in Table 1.

**Table 1:** Demographic data

Category	Variable	*N* [= 375] (%)
Age	Mean [years] ± SD	50,30 ± 16,16
	Minimum [years]	18
	Maximum [years]	88
Gender	Female	204 (54.5)
	Male	169 [45.2)
	Divers	1 (0.3)
	No answer	1
Migration background	Migrants	73 (19.5)
	Natives	302 (80.5)
Marital status	Single	63 (16.9)
	Married	233 (62.5)
	In a partnership / Cohabiting	38 (10.2)
	Divorced	30 (8)
	Widowed	9 (2.4)
	No answer	2
Residence	Metropolis	14 (3.8)
	Rural area	353 (96.2)
	No answer	8
Religion	Christian	265 (71.2)
	Muslim	41 (11)
	Other	4 (1.1)
	Atheistic/ no religion	62 (16.7)
	No answer	3
Educational background	No school diploma/ graduation	3 (0.8)
	Lower secondary school (Hauptschule, Germany)	141 (39)
	Intermediate secondary school (Realschule, Germany)	129 (35.6)
	General university entrance qualification (Abitur, Germany) / University of applied sciences entrance qualification (Fachhochschulreife, Germany)	46 (12.7)
	University degree	43 (11.9)
	No answer	13
Number of children	No children	104 (27.8)
	One	64 (17.1)
	Two	134 (35.8)
	More than two children	72 (19.3)
	No answer	1
Chronical disease	None	180 (49.6)
	≥ 1^a^	183 (50.4)
	No answer	12

(SD= standard deviation, n= number, ^a^chronical disease= Asthma, cardiac arrhythmia, and diabetes mellitus)

### Participants with a migration background

Of the 375 respondents, 19.5% (*n* = 73) reported a migration background. Table 2 provides an overview of their demographic characteristics, including country of birth, German language proficiency, general health status, and length of residence in Germany. Nearly half were born in Germany (47.9%), while others originated mainly from Turkey (21.1%). Most participants indicated good to excellent German language skills and a comparable level of general health. Most participants with a migration background had lived in Germany for more than 20 years (80.8%). The mean age of this subgroup was 45.4 years (SD 15.2), indicating that the study population largely consisted of long-term migrants.

**Table 2:** Characteristics of participants with a migration background

Category	Variable	*N* [= 73] (%)
Country of birth	Germany	34 (47.9)
	Turkey	15 (21.1)
	Poland	6 (8.5)
	Hungary / Lithuania / Pakistan / Romania^a^	8 (11.3)
	Bosnia-Herzegovina / Brasil / Croatia / Italy / Kazakhstan / Kyrgyzstan / Serbia / USA^b^	8 (11.3)
	No answer	2
Knowledge of German	Excellent	24 (33.3)
	Very good	16 (22.2)
	Good	19 (26.4)
	Sufficient	10 (13.9)
	Poor	3 (4.2)
	No answer	1
General state of health	Excellent	3 (4.2)
	Very good	10 (14.1)
	Good	38 (53.5)
	Sufficient	16 (23)
	Poor	4 (5.6)
	No answer	2
Resides in Germany [in years]	1–5	1 (1.4)
	5–10	4 (5.5)
	10–20	9 (12.3)
	≥ 20	59 (80.8)

(^a^= two patients per country, ^b^= one patient per country. Percentage may not sum to 100% due to rounding)

### Comparison of TCIM use between migrants and natives

**Fig. 1:**
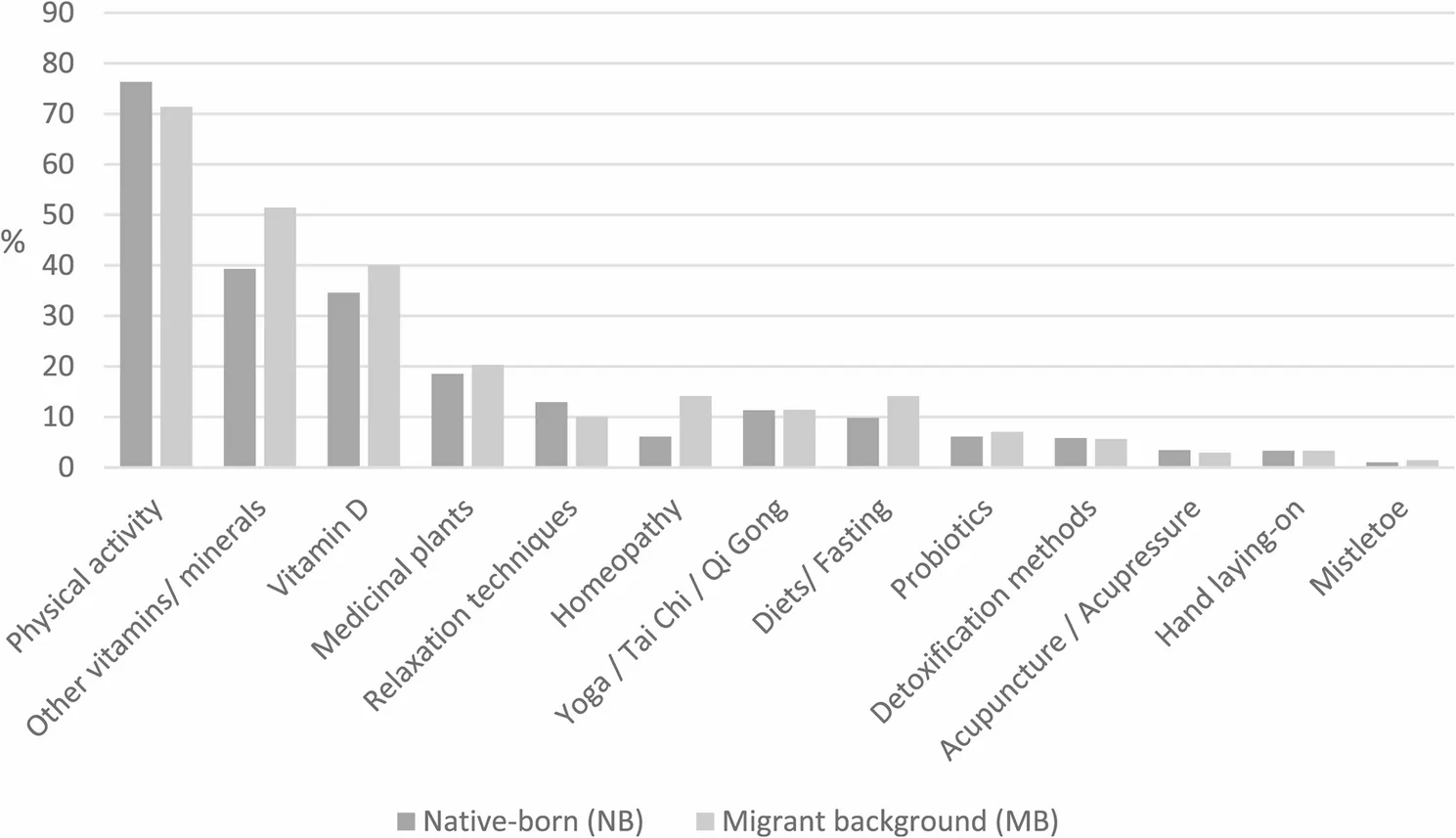
TCIM usage by methods: Native-born (NB) vs. Migrant background (MB)

Overall, 89.5% of participants reported the use of at least one TCIM modality, while 10.5% reported no TCIM use. Regarding the breadth of TCIM use, nearly half of participants applied one to two different TCIM modalities (47.3%), whereas 42.2% reported the use of three or more modalities, indicating a substantial subgroup with broader TCIM engagement. In the sample (*N* = 375), TCIM methods were used to varying extents (Fig. 1). The most frequently reported practices were physical activity (75.2%; NB: 76.3%, MB: 71.4%), the use of other vitamins and minerals (41.6%; NB: 39.3%, MB: 51.4%), and vitamin D supplements (35.6%; NB: 34.6%, MB: 40.0%). Moderate usage rates were observed for medicinal plants (18.8%; NB: 18.5%, MB: 20.3%), relaxation techniques (12.3%; NB: 12.9%, MB: 10.0%), and homeopathy (7.7%; NB: 6.1%, MB: 14.1%). All remaining TCIM methods were used less frequently (each < 15%, most < 5%).

Bivariate chi-square analyses showed no significant differences between NB and MB participants in the use of most TCIM methods (all *p* > 0.05), with consistently small effect sizes (Cramer’s V ≤ 0.10). A significant association was found exclusively for homeopathy, which was more frequently used by MB participants (χ²(1) = 7.27, *p* = 0.007, Cramer’s V = 0.14). A marginal, non-significant trend was observed for the use of other vitamins and minerals, which tended to be more common among MB participants (*p* = 0.065).

Multivariate logistic regression analyses were conducted for TCIM methods with sufficient prevalence (vitamin D, other vitamins/minerals, medicinal plants, and physical activity). For vitamin D, the model was significant (χ²(7) = 25.29, *p* < 0.001; Nagelkerke R² = 0.095), but migration status was not a predictor (OR = 0.81; 95% CI [0.45, 1.46]). Gender emerged as a significant predictor, with women showing higher odds of use (OR ≈ 2.81; *p* < 0.001). For other vitamins and minerals, the model explained 11.1% of the variance (Nagelkerke R² = 0.111), with migration status (OR = 0.53; 95% CI [0.29, 0.95]; *p* = 0.032), educational level (OR = 0.78; *p* = 0.024), and female gender (OR ≈ 2.81; *p* < 0.001) emerging as significant predictors. For medicinal plants, the model narrowly missed significance; migration status was not associated with use, and female gender was the only significant predictor (OR ≈ 2.30; *p* = 0.006).

Additional regression analyses indicated that women were also significantly more likely to use detoxification methods (Exp(B) = 4.55; *p* = 0.018) and hands-on healing practices (Exp(B) = 11.57; *p* = 0.039). For physical activity, higher educational level was the only significant predictor (Exp(B) = 0.64; *p* = 0.006). Neither gender, migration status, nor age group was associated with use. Across all adjusted models, migration status and age group did not emerge as consistent predictors of TCIM use. Model fit indices ranged from low to moderate (Nagelkerke R² = 0.05–0.31).

Taken together, the findings suggest that TCIM use patterns are largely comparable between NB and MB participants. Differences by migration status were limited to homeopathy and, to a lesser extent, other vitamins and minerals, whereas gender consistently emerged as the most influential predictor across multiple TCIM modalities.

### Religious Orientation (RO) according to sociodemographic characteristics

Table 3 summarizes the distribution of the five Religious Orientation (RO) items according to gender and age. For exploratory purposes, the five investigator-developed items were summarized into a composite measure of overall religious orientation. The five-item composite demonstrated excellent internal consistency (Cronbach’s α = 0.92). Although the items were investigator-developed and do not constitute a validated questionnaire, the composite was used as an exploratory measure of overall religious orientation. RO increased across age groups, from the lowest values among participants aged 18–39 years (M = 1.99, SD = 0.83) to the highest among those aged 76 years and older (M = 2.91, SD = 0.68). Women reported higher RO scores than men (M = 2.36 vs. 2.09), and participants with a migration background had higher RO scores than native-born participants (M = 2.57 vs. 2.16). Logistic regression confirmed that higher RO scores were associated with migration background (OR = 1.64, *p* < 0.001). Religious affiliation was strongly associated with all five individual RO items (all *p* < 0.001; Cramer’s V = 0.18–0.28). Participants with a migration background also reported higher scores across these items (all *p* ≤ 0.012; Cramer’s V ≈ 0.17–0.21). Multinomial regression analyses of individual RO items, including “time for thoughts and prayers” and “feeling the presence of God”, showed that religious affiliation was the only consistent predictor (both *p* < 0.001), whereas age, gender, education, and migration background showed no stable independent associations.In contrast, TCIM use (dieting/fasting, acupuncture/acupressure, homeopathy, other TCIM modalities) showed no meaningful associations with religious variables (all *p* > 0.18; Cramer’s V < 0.12), except for a statistically fragile link between homeopathy and the belief that religion influences lifestyle (*p* = 0.032), which was limited by small expected cell counts. Logistic regression models predicting the use of vitamin D, other vitamins/minerals, and herbal remedies showed low explanatory power (Nagelkerke R² = 0.055–0.118), and neither religious nor sociodemographic variables emerged as significant predictors.

**Table 3:** Mean scores of religious orientation items by gender and age group

Religious orientation items	Women	Men	18–39 years	40–59 years	60–75 years	≥ 75 years
Items reflecting intrinsic aspects of religious orientation						
It is important to me to spend time in private reflection and prayer.	2.94	2.59	2.65	2.83	2.90	3.06
I often have a strong sense of God’s presence.	2.42	2.03	2.11	2.30	2.42	2.69
My entire outlook on life is based on my religion.	2.49	2.29	2.33	2.44	2.51	2.63
Items reflecting extrinsic aspects of religious orientation						
I pray mainly to obtain relief and protection.	2.66	2.37	2.43	2.58	2.67	2.81
What religion offers me most is comfort in times of need and sorrow.	2.74	2.46	2.52	2.69	2.76	2.94

Footnote: The grouping into intrinsic and extrinsic aspects is intended solely to facilitate interpretation of the individual items. These categories do not represent validated subscales and were not analysed separately

Overall, the findings demonstrate that religious orientation varied according to age, gender, and migration background, whereas religious affiliation emerged as the most consistent predictor of the individual RO items. Neither religious orientation nor religious affiliation was consistently associated with TCIM use.

### Determinants of health literacy and their associations with TCIM use

Overall, health literacy levels were high (Table 4), with only minor differences across sociodemographic groups, including age, gender, and migration background. Men reported higher values for knowing causes of complaints (χ²(6) = 15.83, *p* = 0.015; V = 0.146; trend *p* = 0.051), knowledge of therapy options (χ²(6) = 12.15, *p* = 0.059; V = 0.128; trend *p* = 0.002), medication indication knowledge (significant), and ability to perform medical treatments at home (χ²(3) = 7.10, *p* = 0.069; Phi = 0.139; trend *p* = 0.023). Education showed the strongest effects, with higher‑educated individuals scoring consistently higher, particularly in personal responsibility (χ²(15) = 39.19, *p* < 0.001; V = 0.188), knowledge of causes (χ²(15) = 23.77, *p* = 0.069; V = 0.147), therapy options (significant), and treatment ability (χ²(15) = 14.19, *p* = 0.511; ordinal trends). Older adults reported higher HL across domains, including personal responsibility (trend *p* = 0.007; V = 0.108), treatment ability (χ²(9) = 18.76, *p* = 0.027; V = 0.130), knowledge of causes (χ²(9) = 24.80, *p* = 0.003; V = 0.149; trend *p* < 0.001), and therapy options (trend *p* = 0.002). Migration status showed mostly nonsignificant differences, though natives tended to score higher in personal responsibility (χ²(3) = 6.54, *p* = 0.088; Phi = 0.132), treatment ability (χ²(3) = 7.10, *p* = 0.069; Phi = 0.139; trend *p* = 0.023), and knowledge of causes (χ²(3) = 6.77, *p* = 0.080; Phi = 0.135).

**Table 4:** Health literacy – descriptive statistics & likert distribution

Item	Mean (± SD)	Not true (%)	Hardly true (%)	Rather true (%)	True (%)
I am personally responsible for my own health	3.49 (0.69)	1.6	6.4	32.9	59.1
The most important thing for my health is that I actively take care of my health.	3.66 (0.55)	0.5	2.1	28	69.3
I am convinced that I can do something myself to prevent illness.	3.56 (0.66)	1.9	3.5	31.2	63.5
I know why I am taking each medication.	3.62 (0.64)	1.6	4	24.8	69.5
I know when to see a doctor and when self-treatment is sufficient.	3.47 (0.67)	1.3	6.1	36.9	55.6
I trust my family doctor, even when my concerns are not directly addressed.	3.61 (0.61)	1.1	3.5	29.1	66.3
I am confident in managing necessary medical treatments at home.	2.92 (0.91)	8.1	20.9	41.7	29.3
I know the causes of my symptoms.	2.90 (0.88)	6.2	26.1	39.8	28
I am aware of the various treatment options for my condition.	2.86 (0.92)	8.1	25.8	37.9	28.2
I have successfully changed my lifestyle (e.g., diet and exercise).	3.11 (0.84)	4.1	18.1	40.3	37.6
I know how to prevent my health from deteriorating.	3.25 (0.68)	1.1	10.3	50.8	37.8
I am confident that I can find solutions if my health deteriorates.	3.10 (0.78)	2.4	18.3	46.4	32.9
I am confident I can change and maintain my lifestyle habits over time.	3.16 (0.74)	1.6	15.3	48.1	34.9

(*SD *standard deviation)

Across the 13 health literacy items, agreement was high (28–69% “true”; 0.5–8% “not true”). Significant differences in favour of natives appeared for “ability to perform medical measures at home” (χ² = 11.69, *p* = 0.009) and “ability to successfully adjust one’s lifestyle” (χ² = 15.22, *p* = 0.002). Strong age effects were found, for example for “knowledge of when to seek medical care” (χ² = 82.86, *p* < 0.001) and “knowledge of therapy options” (χ² = 19.71, *p* < 0.001). Women scored higher in several items, including “ability to perform medical measures at home” (χ² = 21.35, *p* = 0.002) and “knowledge of therapy options” (χ² = 13.49, *p* = 0.036). No stable effects emerged for religion or education in these models.

Bivariate analyses revealed only weak and selective associations between individual health literacy items and TCIM use. Successful lifestyle adjustment was strongly associated with physical activity (χ² = 65.827, *p* < 0.001; Phi = 0.428) and showed weaker associations with Yoga/Tai Chi/Qi Gong and relaxation techniques. Knowledge of medication indications was associated with vitamin D use, whereas the remaining associations were inconsistent and of small effect size.

Binary logistic regressions indicated limited predictive value of HL for TCIM use. In addition, multinomial logistic regression models confirmed the same pattern, showing that only selected HL facets contributed meaningfully to TCIM behaviour. Significant predictors included lifestyle adjustment for physical activity (B = − 1.214, *p* < 0.001; Exp(B) = 0.297) and diets/fasting (B = –0.864, *p* = 0.015; Exp(B) = 0.421); medication indication knowledge for laying on of hands (B = 1.332, *p* = 0.020; Exp(B) = 3.789) and homeopathy (B = 1.067, *p* = 0.031; Exp(B) = 2.908); and knowing when to seek medical care (B = 0.464, *p* = 0.024; Exp(B) = 1.591) and treatment ability (B = − 0.309, *p* = 0.044; Exp(B) = 0.734) for use of other vitamins/minerals. No significant associations emerged for vitamin D, herbal medicine, detoxification, acupuncture/acupressure, or Yoga/Tai Chi/Qi Gong.

## Discussion

In this exploratory cross-sectional study, TCIM use was common among primary care patients and showed only minimal differences by migration background. The high overall prevalence of TCIM use in our sample should be interpreted in light of a broad operationalization of TCIM, which included lifestyle-related practices such as physical activity and relaxation techniques. From a public health perspective, these findings suggest that TCIM use is less a function of cultural or religious background than of broader social determinants such as gender, education, and health literacy. While participants with a migration background displayed significantly higher religious orientation and more frequent religious practices, neither religious orientation nor religious practice was systematically associated with TCIM use. Instead, gender and educational level emerged as the most consistent predictors across TCIM categories, aligning with established social determinants of health. Health literacy was generally high in both groups and showed only selective, method-specific associations with TCIM use.

Our results show no significant difference between native-born and migrant populations in terms of TCIM use, and are thus consistent with other studies from the European healthcare system. The use of TCIM is primarily influenced by sociodemographic factors such as gender, educational level, and subjective health beliefs, rather than by ethnic origin or migration status itself [14]. The higher use of homeopathy among migrants, as described in the results section, is an exception and may be culturally determined, but does not reflect a general trend. In contrast, international studies, for example from the US and Australia, show a higher use of TCIM among migrants, which can be attributed to limited access to conventional care or an affinity for traditional medical systems [15–17]. In populations with a high level of integration, long periods of residence, and good language skills, usage patterns are similar to those of the native population [15].

Gender-specific health behaviors and role models have a significant influence on TCIM use: Women demonstrate greater health awareness, seek preventive and holistic approaches more frequently, and are more open to non-conventional therapies [18, 19]. They rate the perceived benefits of TCIM (e.g., wellness, prevention, control over their own health) more highly and are more receptive to recommendations from social networks [19, 20]. In addition, women are more willing to reveal TCIM use to medical professionals [21].

Our results did not confirm a linear model in the sense of “the higher the level of education, the more TCIM.” Although many studies show that people with higher levels of education tend to use certain TCIM methods more frequently, this correlation is not linear and varies depending on the TCIM category and individual decision-making processes [22, 23]. Decision-making processes also appear to differ according to educational level. Individuals with higher educational attainment tend to prefer evidence-based or lifestyle-related approaches, such as yoga, meditation, or dietary modifications, whereas those with lower educational attainment more frequently use supplement-based or traditional approaches, including vitamins, minerals, or herbal preparations [22, 23]. Usage patterns are also influenced by factors such as health status, personal values, worldview, and expectations of benefit [24]. A lack of health knowledge is a common reason for not using TCIM among people with lower levels of education [25].

A stronger religious orientation may support coping with illness, particularly through prayer and religious practices. Importantly, in this well-integrated migrant sample, religiosity functioned primarily as a psychosocial resource rather than as a driver of health behavior or TCIM use. International studies show that such religious resources are often used as emotional coping strategies and are sometimes classified as TCIM-related practices [26, 27]. However, in well-integrated migrant samples—such as the one in this study—there are no stable correlations between general religiosity or religious practices and the use of complementary methods. This suggests that spiritual resources are relevant for coping but are not necessarily correlated with TCIM methods in the narrower medical sense (e.g., naturopathy, manual therapies, supplements) [26, 27]. Prayer and spiritual practices may also function as independent coping strategies and therefore do not necessarily predict TCIM use [28]. Beyond prayer and other religious practices, TCIM use itself may also represent an individual coping strategy in response to chronic illness. However, coping was not directly assessed in the present study, and this interpretation should therefore be considered hypothesis-generating and warrants further investigation. Furthermore, the analyses showed that specific religious practices—such as prayer or experiencing divine presence—could be explained almost exclusively by religious affiliation, while migration, age, or education played hardly any role. These findings suggest that the study-specific RO items consistently captured related aspects of religious orientation within our study population, supporting the internal consistency of the measure.

The relationship between HL and the use of TCIM is complex and population-dependent. In the present sample, which had a high overall level of HL, only selective and predominantly weak correlations with TCIM use were found, meaning that HL is not a consistent predictor of TCIM [29, 30]. People with high HL tend to use TCIM in a targeted, critical, and often evidence-based manner [29, 31]. At the same time, HL has been shown to influence self-efficacy, successful lifestyle changes, and active disease management. Meta-analyses show that high HL is a key prerequisite for effective self-management, especially in chronically ill and older people, with the effect being partly direct and partly mediated through increased self-efficacy [32, 33]. In vulnerable groups with low health literacy, on the other hand, uncritical or risky use of certain forms of TCIM, such as herbal preparations, dietary supplements, or traditional practices, is more common [34]. Here, limited information and evaluation skills make evidence-based decision-making difficult and encourage unreflective use of TCIM.

Our data indicate that religiosity, health literacy, and migration background are related but largely independent dimensions of health-related behavior. TCIM use appears to be shaped predominantly by social determinants such as gender and education rather than by cultural or religious orientation. These findings support a public health approach that prioritizes equity-oriented, non-stereotypical care and addresses structural determinants of informed health decision-making. TCIM use appears to be shaped less by cultural or spiritual orientation than by individual sociodemographic factors, particularly gender and education, which aligns with broader public health evidence on behavioral determinants. Recognizing this differentiation may help clinicians address patients’ spiritual resources without overinterpreting their relevance for therapeutic preferences.

### Limitations and future directions

This study is limited by its cross‑sectional design, which precludes causal inference, and by its conduct in a single, predominantly rural primary care setting. The migrant subgroup was relatively small and largely composed of individuals who had lived in Germany for many years, indicating a well‑integrated population. Migration background was assessed only at a broad level and did not capture factors such as country of origin, age at migration, or migration generation, all of which may shape health beliefs and TCIM use. This likely reflects recruitment from a single established practice with long‑term patient relationships. As a result, the findings cannot readily be generalized to recently arrived migrants or individuals with lower levels of social integration, whose healthcare utilization and TCIM patterns may differ. At the same time, the sample represents a subgroup commonly encountered in German primary care and is therefore relevant to the research question.

Multiple exploratory analyses were conducted without formal adjustment for multiple comparisons, meaning that some statistically significant results may represent chance findings and should be interpreted cautiously. TCIM use was assessed in broad self‑reported categories without information on frequency, motivations, or perceived effectiveness, and all variables were collected by self‑report, making the data susceptible to reporting bias, including social desirability and recall bias. This may have particularly affected responses related to acculturation, religious orientation, and health‑related behaviours. The interpretation and use of specific TCIM modalities—such as vitamin D supplementation—may also be shaped by differences in healthcare systems, prescribing practices, and preventive care recommendations across countries of origin, which were not assessed.

Religious orientation was assessed using investigator-developed items rather than a validated multidimensional questionnaire. Although the five-item composite showed excellent internal consistency, future studies should use validated instruments to distinguish between different dimensions of religious orientation. Religious affiliation was assessed only at a broad level, without differentiation between Christian denominations or wider dimensions of spirituality, limiting the ability to examine potentially relevant differences in religious traditions and spiritual beliefs. Future studies should employ instruments that distinguish more clearly between religious orientation, spirituality, and denominational affiliation and should use longitudinal or mixed‑methods designs with more diverse migrant samples to better disentangle their roles in health behaviour and TCIM use.

## Conclusion

This study demonstrates that TCIM use is common in primary care patients but is largely independent of migration background and religious orientation. Although migrants exhibited higher levels of religiosity and religious practice, these factors did not translate into distinct patterns of TCIM utilization. Instead, female gender and educational level emerged as the most consistent predictors of TCIM use, while health literacy showed only selective, method-specific associations. These findings suggest that religiosity primarily functions as a coping and meaning-making resource rather than a determinant of therapeutic behavior. For culturally sensitive care, these results underscore the importance of avoiding stereotypical assumptions and instead prioritizing individual health beliefs, competencies, and preferences. Moreover, the findings highlight the need to address broader social determinants—particularly gender and education—when designing public health strategies aimed at supporting informed and equitable health decision-making.

## Data Availability

The dataset supporting the conclusions of this article is included within the article.
